# Mapping the Transport Kinetics of Molecules and Particles in Idealized Intracranial Side Aneurysms

**DOI:** 10.1038/s41598-018-26940-1

**Published:** 2018-06-04

**Authors:** Mark Epshtein, Netanel Korin

**Affiliations:** Technion- Israel Institute of Technology, Department of Biomedical Engineering, Haifa, 32000 Israel

## Abstract

Intracranial side aneurysms (IA) are pathological blood-filled bulges in cerebral blood vessels. Unlike healthy blood vessels where mass transport is dominated by convection, both diffusion and convection can play an active role in aneurysm sites. Here, we study via dye washout experiments and numerical simulations, the transport characteristics of particles (1 micron) and small molecules (300 Da) into simplified side aneurysms models following bolus injection. Time-lapse fluorescent microscopy imaging performed in our idealized aneurysm models showed that the parent artery geometry (located on the inner vs. outer curvature) as well as the aneurysm aspect ratio (AR) affect the washout kinetics while the pulsatile nature of the flow, maintained within the physiological range, carries only a minor effect. Importantly, in the absence of effective diffusion, particles that are located on slow streamlines linger within the aneurysm cavity, a phenomenon that could be of importance in deposition of cells and nano/micro-particles within aneurysms. Altogether, mass transport studies may provide valuable insights for better understanding of aneurysm pathophysiology as well as for the design of new diagnostic and theranostic nano-medicines.

## Introduction

Intracranial aneurysms (IA) are pathological blood-filled sac like bulges in cerebral blood vessels. IA occur in about 5% of the population where 0.2% rupture with a mortality rate of nearly 50%^1,2^. Progression and the rupture of IA have been associated with the presence of inflammatory processes, atherosclerotic plaque formation and blood clots on the aneurysm wall^3,4^. Such processes are tightly related to local hemodynamics and mass transport characteristics. Hemodynamic forces in general, and shear stress in particular, have been shown to affect endothelial cell physiology where abnormal fluidic shear stress profiles result in a pro-inflammatory phenotype contributing to disease progression^2,5–8^. This link between hemodynamic forces and endothelial dysfunction have been the focus of many studies analyzing the wall shear stress profiles in aneurysms, in a patient specific manner, via computational fluid dynamic (CFD) simulations with the aim of correlating it with disease progression and risk of rupture^2,9–11^.

In addition to affecting the shear stress exerted on endothelial cells, abnormal blood flow also affects mass transport at disease sites. Hence there has been an increased interest in studying the abnormal mass transport in aneurysms with particular focus on bio-molecules that can play a role in disease progression such as ATP, LDL and oxygen transport^7,12,13^. One of the most important parameters that determine the nature of mass transport is the Peclet number (Eq. 1), defined as the ratio between the rate of advection to the rate of diffusion, see Eq. 1- where *L* is a characteristic length scale, *u* characteristic velocity and *D* is mass diffusivity coefficient. Under normal blood flow, *Pe* number is relatively high (*Pe* > 1) and mass transport is governed by advection. However, inside a side aneurysm cavity the flow can be several orders of magnitude slower resulting in a lower local Peclet numbers (*Pe* ≤ 1). Thus, in sites of aneurysm, mass transport is a complex convection-diffusion problem where both convection and diffusion play a role.

1 $${Pe}={uL}/{D}$$

Besides the transport of blood borne molecules, another important convection-diffusion problem that has been studied widely is the transport of contrast agents into aneurysms. CT angiography, based on X-ray imaging during the injection of an iodine-based contrast agent, which are typically small-molecule (<2,000 Da)^14^ is a standard routinely used procedure in aneurysm diagnosis and monitoring. The transport of contrast agent can provide valuable information on the examined aneurysm and thus can be useful in treatment planning as well as in evaluating the performances of flow altering procedures, such as coil embolization^15^. Additionally, “virtual angiography” performed via numerically solving the convection–diffusion equations describing bolus dye injection have been suggested as a method to validate patient specific CFD models of aneurysms^16,17^.

Recently, there has been growing interest in the use of particulate (e.g.; nano carriers) as imaging agents, and even potentially as theranostic agents, that can offer an improved signal as well as specific molecular targeting capabilities (e.g.; molecular imaging of inflammation)^18–20^. As outlined above, there have been a variety of computational studies focused on the transport of small molecules into side aneurysms, however, the subject of particulate transport into aneurysm received so far little attention. Furthermore, current research of material transport in aneurysms sites is mainly numeric^21,22^. Numerical studies are very useful and allow basic understanding of the studied phenomena but require validation and are limited in their scope due to computational limitation.

Here, we study experimentally and numerically, via dye washout experiments, the transport of particles (1 micron) and small molecule (approx. 300 Da) into simplified side aneurysms models following bolus injection. As hemodynamics and transport properties are governed by aneurysm geometry, we explore aneurysms of different aspect ratios (aneurysm/vessel diameter *AR* = 1.6 and 3.2) and at different parent artery geometries (inner/outer curvatures). The latter is known to affect the local Dean number defined as the ratio between the square root of the product of centripetal and inertial forces to the viscous forces, see Eq. 2 where *d* is the parent artery diameter,*r* is its radius of curvature and *Re* is the Reynolds number^23^.

2 $$De=Re\sqrt{\frac{d}{2r}}$$

Additionally, as the pulsatility index (PI) of blood flow, defined as the ratio of the difference between the maximum and the minimum Reynolds numbers to the mean Reynolds (Eq. 3), may affect hemodynamics and mass transport^24^, we explore the effect of different PI values and compare it with the case of a constant flow profile.

3 $$PI=\frac{R{e}_{max}-R{e}_{min}}{R{e}_{mean}}$$

Altogether, our result may shed light on the transport characteristics of particulate and small molecules into aneurysms and provide valuable information for better understanding of aneurysms pathophysiology as well as for the design of new diagnostic and theranostic nano-medicines.

## Results and Discussion

**Figure 1:**
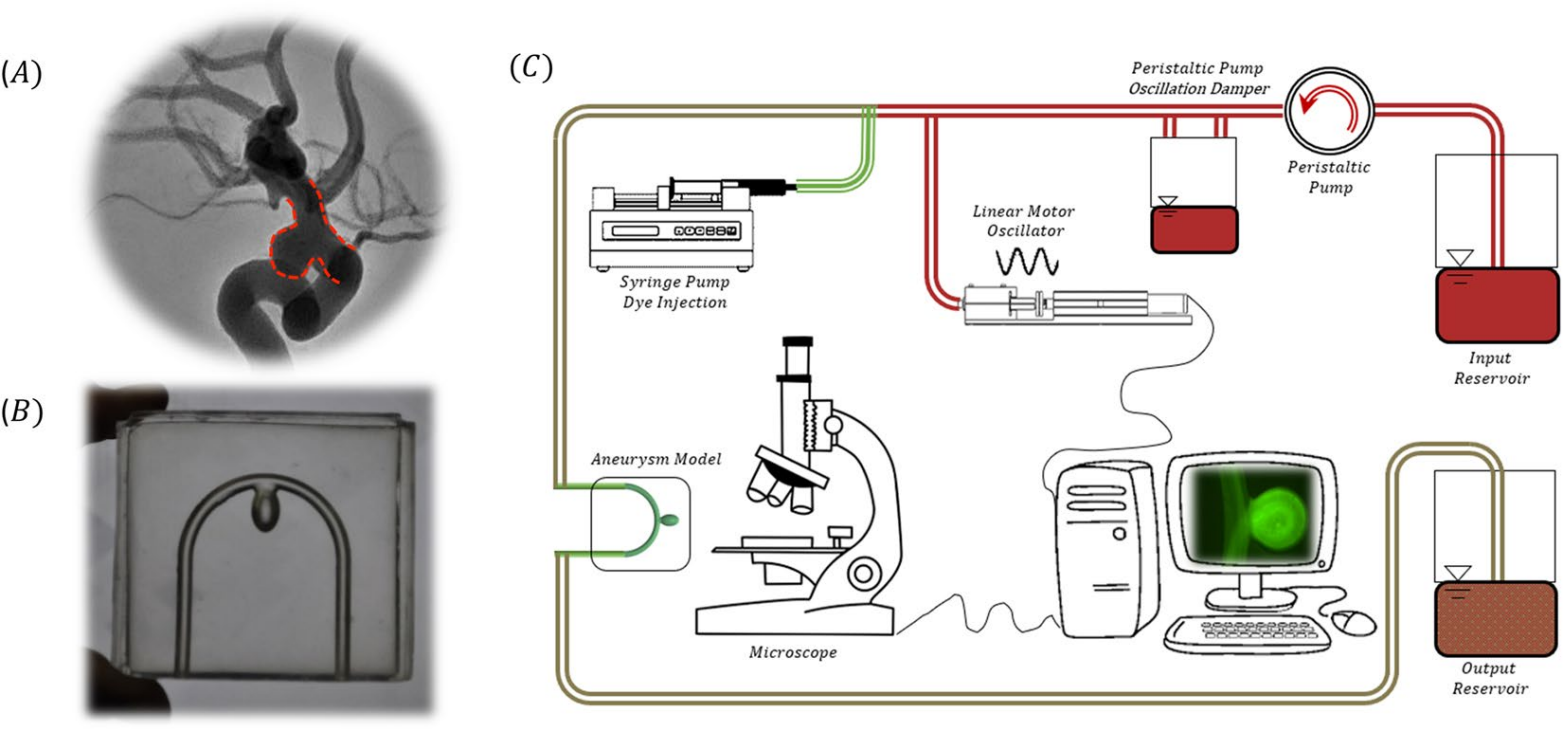
Experimental setup for studying transport kinetics into a side aneurysm model. **(A)** A representative cerebral side aneurysm CT angiography. Aneurysm borders are marked in red. (**B**) Transparent silicone aneurysm phantom model (3.2 AR, inner geometry) produced via 3D printing and silicon molding. (**C**) Schematic of the experimental system for real-time microscopy imaging following a bolus injection of a fluorescently tagged solution. A custom designed perfusion system allows the emulation of a blood flow waveform via periodic disruption of a steady flow.

The mass transport into side aneurysms was examined both experimentally and numerically, focusing on the effect of aneurysm physiological parameters on both dye and particle transport into the aneurysm cavity. 1-micron particles were used to models nano/micro-particle transport while a Fluorescein molecule (M.W. 300 Da) was used to model small molecules (e.g.; iodinated contrast agents). To study the effect of geometry we fabricated simplified aneurysm models with two representative aspect ratios (AR1.6 & AR 3.2), located on both an inner curvature (IN) and an outer curvature (OU). The models’ parent artery curvature correlate with a Dean number of 120 (>75, critical Dean number in a tube) which implicate a non-negligible effect of centrifugal forces on the flow^23^, see *Methods* section and *Support Materials* for details on the geometries. The examined models were connected to a custom-built perfusion system, placed horizontally under a microscope and time-lapse fluorescence microscopy imaging was performed to study the transport of either dye or particles into the aneurysm cavity following bolus injection, as shown in Fig. 1, see *Methods* section for details on experimental and imaging procedures.

**Figure 2:**
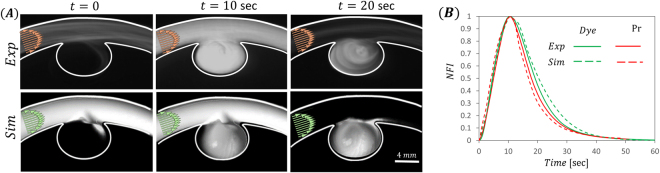
Experiments and simulations of fluorescence intensity maps and corresponding normalized fluorescence intensity (NFI) curves illustrating dye transport following bolus injection. (**A**) Top: Representative experimental results showing time series fluorescence intensity maps following bolus injection of a dye solution. Maps show the filling and washout of dye in the aneurysm cavity for the presented 1.6 AR inner (IN) geometry. Bottom: Simulation results showing time series fluorescence intensity maps following bolus injection of the dye solution for the same aneurysm as in A. (**B**) Normalized fluorescence intensity (NFI) curves within the aneurysm cavity for the corresponding model. Note the experimental (solid line) and simulation (dashed line) results show good agreement for both and dye (curves in green) and particles (curves in red) injections.

Simulations, using a convective-diffusion model, were performed on the same geometrical models, see Methods section for details. Figure 2A shows representative results of the florescence intensity maps observed following bolus injection of a fluorescent dye as well as its corresponding simulation results, Fig. 2B. The figure shows a qualitative similarity between simulation and experimental bolus dynamics. To quantify the filling and washout kinetics within the different aneurysms, we define a region of interest which covers the aneurysm cavity and measure the average fluorescence intensity within the ROI as a function of time. Figure 2C presents the Normalized Fluorescence Intensity (NFI) curves of both dye and particles for the aneurysm present in Fig. 2A,B. As the aneurysm fills the curves rises and reaches its maximum value. From this point on, the intensity decreases, representing the washout kinetics of the dye/particles.

### Effect of aneurysm geometry on dye and particle transport kinetics

**Figure 3:**
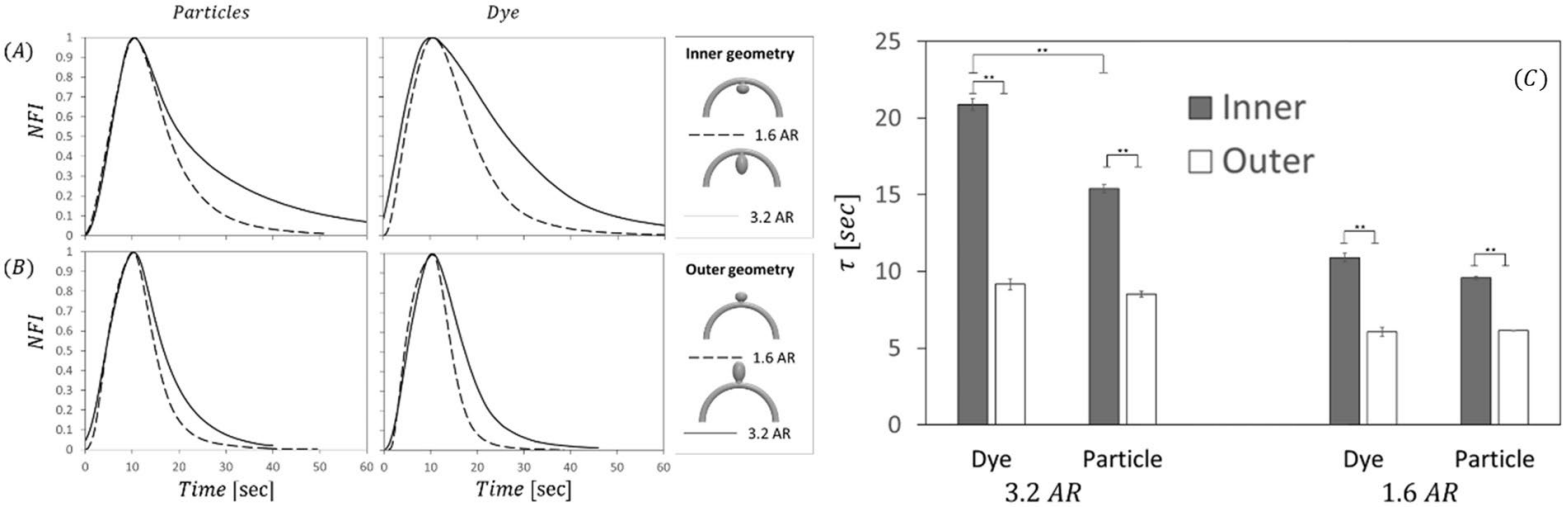
Aneurysm geometry effect on the normalized fluorescence intensity (NFI) curves, following either dye or particle injections. (**A**) Inner geometries with 1.6 AR (dashed line) compared to 3.2 AR (solid line). Left: particle injection, right: dye injection. (**B**) Outer geometries with 1.6 AR (dashed line) compared to 3.2 AR (solid line). Left: particle injection, right: dye injection. (**C**) Bar graph summarizing the time of clearance (τ) measured for the various geometries. Note that in all cases, clearance time for inner and outer geometries of the same AR are significantly different (p < 0.01, t-test). Interestingly, for the 3.2 AR inner aneurysm the particle clearance time is significantly lower (by approx. 25%) compared to the dye’s clearance time.

Figure 3 shows the NFI curves of the examined geometries following bolus injection of dye or particles solutions. Although all of the curves representing the different geometries in Fig. 3A show a similar time of filling the cavity (approx. 10 +/− 0.4 sec), washout kinetics differ between the geometries. To characterize the washout kinetics, we define a signal decay time constant (τ) as the time it takes the curve to drop from the maximum fluorescence to 37% (1/e). As expected, aneurysms with larger AR show slower washout kinetics and the time constant between aneurysm with AR1.6 and AR 3.2 increases by more than 35%, see data in Fig. 3C.

Additionally, the result of Fig. 3C shows that the parent vessel geometry has a significant effect on the washout time of both particles and dye, as evident by τ values that are significantly larger for inner geometries compared outer geometries with the same aspect ratio. These results are in agreement with the value of the Dean number in our system (De = 120), highlighting that centrifugal forces are significant. Accordingly, the aneurysms located on the outer curvature of the parent artery are exposed to enhanced convection facilitated via centrifugal effects. On the other hand, the inner aneurysm geometry isolates the aneurysm from the centrifugal effects and thus reduces the washout compared to the outer geometry^12,25,26^.

**Figure 4:**
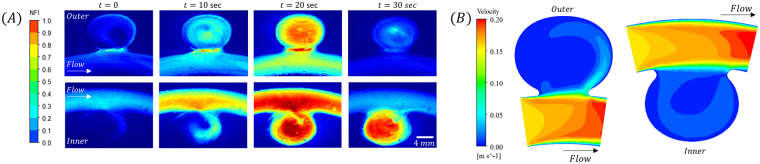
Parent artery geometry effect on aneurysm transport kinetics. (**A**) Time series of fluorescence intensity maps in 1.6 AR inner (up) and outer (down) models showing parent artery and aneurysm intensity difference produced due to skimming during filling of the aneurysm. Note that the intensity within the aneurysm cavity at the outer model can become higher than in the parent artery (*t* = 20 sec). (**B**) Corresponding velocity contour map on the symmetry plane for the outer and inner aneurysms with 1.6 AR geometries, illustrating the differences in convective flux between the outer geometry and the inner geometry. Note that the outer geometry exhibits enhanced convection into the cavity.

To illustrate the different transport kinetics between inner and outer geometries we present in Fig. 4 color-bar fluorescence scaled time-lapse images of the same aneurysm in an outer configuration (Fig. 4A, top panel) compared to an inner configuration (Fig. 4A, bottom panel), following bolus injection of a dye. The results show that, unlike the inner aneurysm case, the intensity within the aneurysm cavity at the outer model becomes higher than in its parent artery. For the inner geometries, centrifugal forces produced by the artery bend, push the bolus material away from the aneurysm increasing the effect of skimming and further reducing fluorescence in the aneurysm^12,25,26^. This is clearly seen in the smaller inner ARs were the parent artery is more fluorescent than the aneurysm while for the outer geometries the effect is reverse and the dye concertation inside the aneurysm becomes actually larger than in the main artery (Fig. 4A). Figure 4B shows the corresponding velocity magnitude field within the inner and outer aneurysm highlighting the difference in the convective flux into the cavity.

### Particles vs. Dye - Particle Lingering within the Aneurysm Cavity

**Figure 5:**
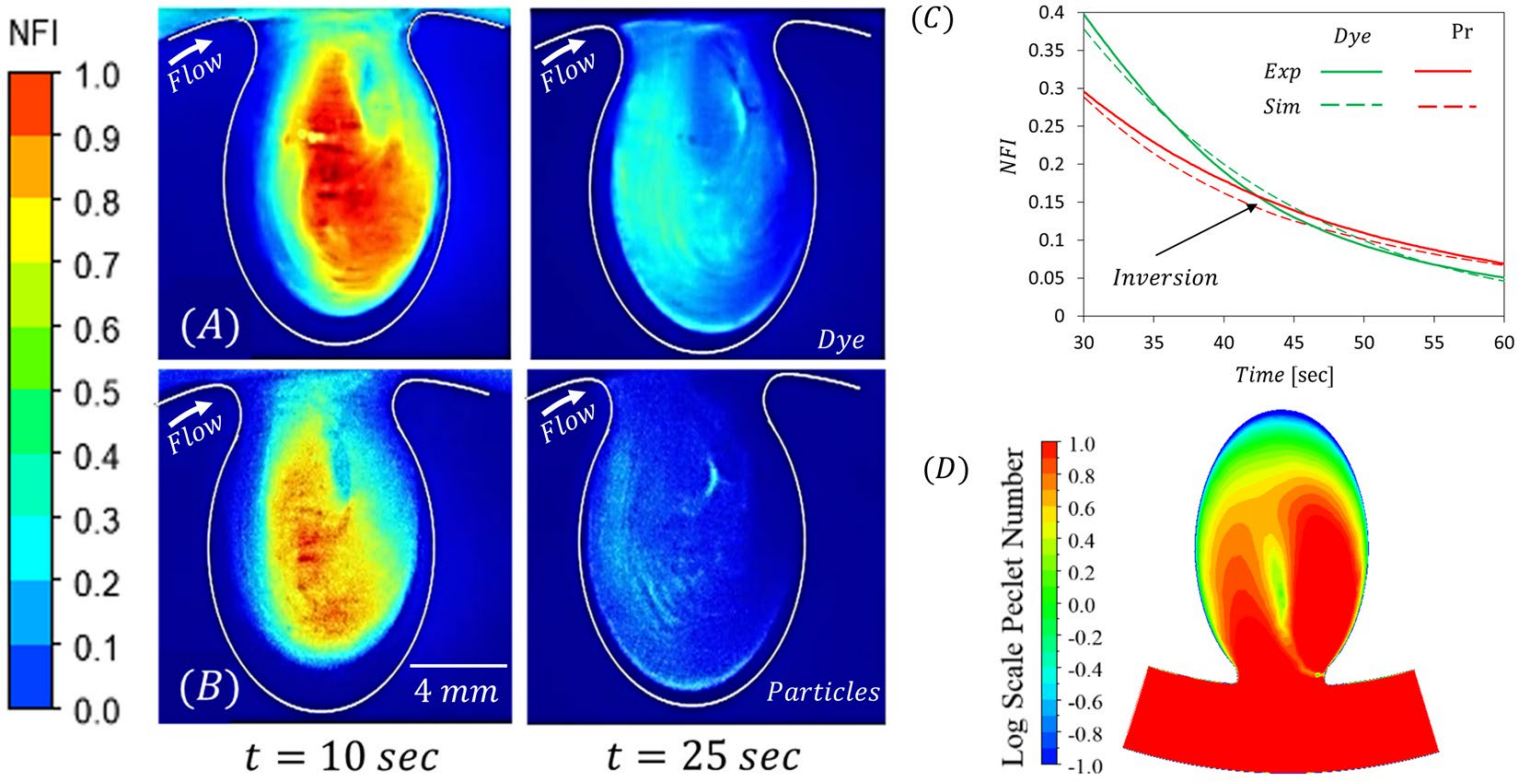
Particle vs dye transport within the aneurysms. (**A**) Time series fluorescence intensity maps following bolus injection of the dye solution. The maps show the filling (*t* = 10 sec) and washout of dye (*t* = 25) in the aneurysm cavity (**B**). Time series fluorescence intensity maps following bolus injection of the particle solution using the same geometry as in A where differences between the dye and the particles can be seen at *t* = 25 sec as particles are observed to focus in certain regions while dye is more diffuse in the cavity. (**C**) NFI curves of 3.2 AR inner geometry showing an inversion point (*t* = 41 sec) – past this time point the NFI of the particles is higher than the dye’s NFI due to particle lingering within the aneurysm cavity, (**D**) Contour map of local Peclet number showing that particle lingering regions in B correlate with regions of low Peclet number (Note: log scale color map).

As shown in Fig. 3B there are pronounced differences between dye and particle washout times at the larger inner (AR = 3.2) aneurysms, where τ shows significantly higher values in the case of particle injection. Interestingly, although more moderately, these differences were also observed when experiments were conducted with an ultra large 2 M Dalton dextran dye, see Fig. 4S and supporting material. Figure 5A shows representative fluorescence intensity maps following the 300 Da dye injection while the bottom panel, Fig. 5B, shows results following particle injection, both in an IN aneurysm with AR = 3.2. Though filling kinetics are similar, see t = 10, washout of the fluorescent signal differs between dye and particles solutions, see t = 25 sec as well as Movie 01 in *SOM*. At this time point, the particles focus in certain regions while the dye images look more diffusive, filling a larger portion of the cavity. Interestingly, when looking at later time points, the NFI curves for dye and particles intersect and flip showing higher particle fluorescence toward the end of the curve (indicated by the arrow Fig. 5C). This is due to the fact that dye that was located at regions characterized by low velocity is washout by diffusing to region with higher convection. However, as particle diffusion is negligible, particles that are located on slow streamlines remain to linger within the aneurysm cavity. As shown in Fig. 5D, these regions where particles linger, correlate with regions exhibiting a low local Pecelt number (*Pe*_*d*_ < 1), where we define *Pe*_*d*_ using the particle diameter as a characteristics length scale, allowing a more relevant *Pe* compared to the conventional *Pe* based on the parent artery diameter.

This effect where particles linger in the aneurysm is seen in all of the studied aneurysm geometries. For example, even in 1.6 AR outer geometry where stasis regions are non-existent, at longer time points the particles curve is above the dye curve and particles are observed lingering in the middle of the central vortex (see SOM Movie 02). The phenomena of particle lingering in stasis regions within aneurysm cavities can be valuable for studying deposition of cells and particles within aneurysm cavities. Altogether, our results illustrate that at sites of aneurysms dye and particles behave differently.

### Effect of parent artery curvature

**Figure 6:**
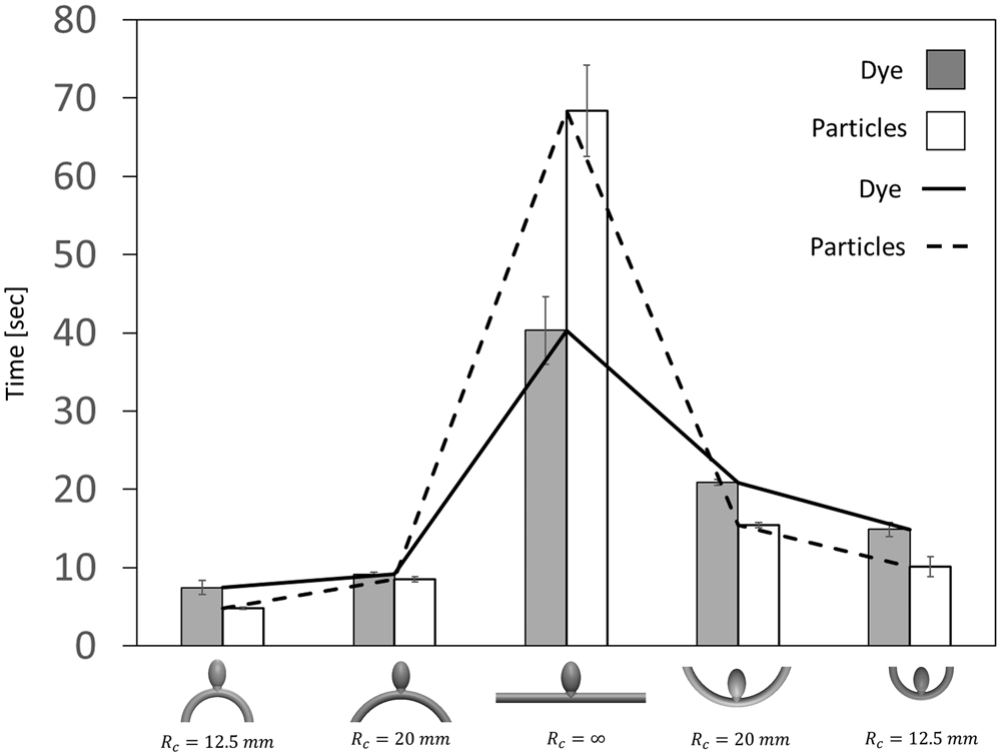
The effect parent artery curvature on dye and particle washout time. Bar graph showing washout time constants of the same aneurysm (3.2 AR) in five different parent artery geometries (curvature radius: *Rc* = −/+12.5 *mm*, −/+20 *mm* and *Rc* = ∞, i.e. straight vessel). A significantly higher clearance time is shown for the straight aneurysm configuration compared to aneurysm models situated on both inner and outer curvatures (i.e. curvature radius: 12.5 *mm* and 20 *mm*).

To further explore the effect of parent artery geometry on mass transport into aneurysms, we measured the washout time constant for aneurysms (AR 3.2) located on five different parent artery curvatures (curvature radius: *Rc* = −/+12.5 mm, −/+20 mm, and *Rc* = ∞, i.e. straight vessel). The results presented in Fig. 6 (and SOM movie 4) show that the straight aneurysm configuration exhibits a significantly higher clearance time compared to aneurysm models situated on both inner and outer curvatures (i.e. curvature radius: 12.5 *mm* and 20 *mm*). For dye, the washout time in the straight artery configuration was 40.3 +/−4.3 *sec* compared to 14.9 +/−0.9 *sec* at *Rc* = 12.5 *mm* and 20.9 +/−0.4 *sec* at *Rc* = 20 *mm* in the inner curvature conformations and 7.4 +/−0.9 and 9.2 +/−0.3 in the corresponding outer curvature configurations. These changes may be attributed to centrifugal forces which produce enhanced mixing via the formation of Dean vortices known to occur at high curvatures, thus allowing an enhanced washout^23^. On the other hand, the straight parent artery geometry produced minimal disturbance of the vortex at the aneurysm, which translated to prolonged particle lingering in the aneurysm cavity and to a significantly longer particle washout time compared to the dye washout time and to the other curved vessels. Altogether, our results illustrate the dominant role of parent artery geometry on aneurysm transport kinetics while opening interesting research possibilities into the connection between aneurysm configuration and prognosis.

### Effect of Flow Pulsatility

**Figure 7:**
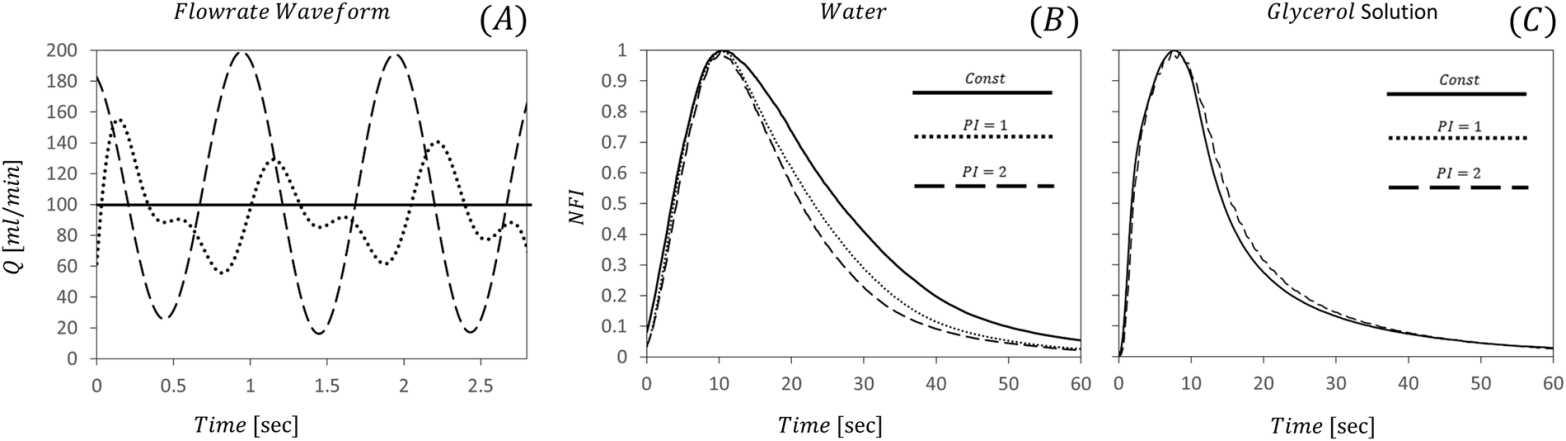
Effect of Pulsatility and medium viscosity on dye transport kinetics. (**A**) The examined input pulsatile waveforms: PI = 2 (dashed line), PI = 1 (dotted line) and constant flow (continuous line). (**B**) Effect of pulsatility index (PI) in an aqueous medium on the NFI curve following dye injections. (**C**) Effect of pulsatility index (PI) in a 4 cP viscous 40% Glycerol-water (mimicking blood viscosity) solution on the NFI curve following dye injection. Note curves almost overlap between constant flow and the PI = 2 waveform.

To investigate the influence of pulsatility on dye and particle transport two pulsatility modes were examined: (1). a *PI* = 2 sinusoidal waveform to insure that pulsatility is above the critical value for vortex breakup [*PI*_*cr*_ =  Parent artery diameter/aneurysm entrance length, in our case *PI*_*cr*_ = 1.04^24^], and (2). a physiological waveform similar to the basilar artery with a pulsatility index of 1^27,28^, See Fig. 7A. Additionally, to further understand the influence of pulsatility under relevant physiological conditions, we also examined a 40% Glycerol and water solution with a viscosity of 3.8 *cP* corresponding to the Newtonian value of the viscosity of whole blood^29^.

Figure 7 presents the results following dye injection into the 3.2 AR IN geometry – see also SOM Movie 03. For the PI = 2 pulse in water, washout kinetics are enhanced significantly by the pulsatile waveform. This enhanced transport may be attributed to the breakup of vortices^24^. Lowering the pulsatility index to 1 reduces the effect markedly, see Fig. 7B & SOM Movie 03. However, importantly, when the medium is changed to a 4 cP solution, mimicking blood viscosity, even a PI = 2 pulse carries no significant effect on the washout curve compared to no pulsatility, see Fig. 7C & SOM Movie 03. This results may be attributed to the differences of the Womersley number between the different viscosities. The Womersely number (eq. 4) is defined as the ratio between the transient inertial forces to viscous forces where L is a characteristic length-scale often chose as the radius of the parent artery *ω* is the angular frecuency *ρ* is the density of the medium and *μ* is the dynamic viscosity of the medium.

4 $$\alpha =L\sqrt{\frac{\omega \rho }{\mu }}$$

Thus, for water and 1 beat per second in the studied 3.2 inner aneurysm the Womersley number is 4.4 indicating a Womersley type flow profile in the pipe with retrograde flow at the aneurysm neck^30^. Increasing the viscosity four-folds lowers the Womersley number, suppressing the retrograde flow and reducing the mixing. Moreover, it has been shown that heat and mass transfer in areas of circulation are not affected much by pulsatility when the Schmidt number is over 1000^31^, as is in our case for both mediums (see supplementary material for detailed calculations). Additionally, it is worth noting that due to the non-Newtonian shear-thinning behavior of blood observed *in vivo*^29^, blood viscosity inside cerebral aneurysms would be higher than in the parent arteries further reducing the role of pulsatility in mass transfer in cerebral side aneurysms^13,31^.

## Conclusion

Our experimental and numeric dye washout results showcase that mass transport properties are highly influenced by aneurysm and parent artery geometries (inner/outer curvatures). The results also suggest that within physiological limits pulsatility carries only minor effect on the filling and washout kinetics. Importantly, we find that small molecules and particles behave differently. To begin with, dye diffuses to regions of low velocities while most of the particles are washed by the main streamlines, at longer times the dye diffuses to faster regions and is cleared faster while particles remain to linger in slow streamlines. This effect of particle lingering within aneurysms maybe valuable for studying and understanding the deposition of cells and particles within aneurysm cavities.

## Methods

### The Experimental Flow System

To emulate a realistic pulsatile flow of cerebral aneurysms a mechanical representation of the Womersley solutions, superimposing an oscillatory flow on a constant flow, was built. Figure 1C shows a schematic of the experimental system. The system consists of two reservoirs, a peristaltic pump (Watson Marlow 530C), an oscillation damper to remove the peristaltic oscillations to produce a steady and constant flow rate (50 *ml/min* for water and 200 *ml/min* for the Glycerol solution) at a Reynolds number of 300 similar to many cerebral arteries^32^, past the damper a syringe is connected to a linear motor (a LinMot® P01-23 linear motor) which connects in parallel to the tubes to produce the oscillatory part of the waveform. The superposition of the constant and the oscillatory flows produces the desired waveform. Downstream of the oscillator a syringe pump (New Era Pumps Systems Inc. NE 1000) is connected and used to inject a bolus of 2.5 *ml* dye or particle solution. To the tubing the aneurysm silicone model was connected and placed horizontality under a microscope for flow visualization.

### Flow Visualization

The flow was visualized using a Nikon SMZ25 upright microscope with x2 magnification. Time-lapse videos were produced using the NIS Elements AR 4.50.00 program. As Dye solution, fluorescein – sodium salt (Sigma-Aldrich,) 5[*mg*/100 *ml*] water solution was prepared. For particle solutions, 1 *μm* fluorescent polymer microspheres (Thermo Scientific™ Fluoro-Max Dyed Red Aqueous Fluorescent Particles) were suspended in water solution at a concentration of 14[*mg*/100 *ml*]. The particle density is 1.05 [*g*/*mm*^3^] producing negligible buoyancy in water. The models were positioned horizontality to discard gravity effects which can influence mass transport, see supporting material for details.

### Aneurysm model fabrication

Computer aided design (CAD) models of the chosen geometries have been drawn and 3D printed from RDG 720 plastic using an Objet Eden 500 printer. The molds were then filled with silicon (Dow-Corning Sylgard®184) mixed with its curing agents (1:10 mass ratio). The cured molds were then placed in an acetone bath overnight to soften the plastic and allow its removal from the silicone cast.

### Simulations

Simulations were performed in Ansys Fluent™ utilizing the heat transfer model while applying the analogy and parametric conversion to mass transfer. The fluid was assumed to behave as a Newtonian fluid with a viscosity of 1 *cP* for water and 3.5 *cP* for blood. The bolus injection was modeled by a custom user defined function which injects a 10 *sec* bolus similar to the experiments. The same CAD models used for the constructions of the silicone models were used also for the simulation and were meshed using Ansys GAMBIT. 60,000 steps with a time step of 0.001 sec was used to produce a 60 second simulation. To emulate the fluorescence difference between the dye and particles, as seen experimentally, the results were fringed to fit best the experimental results.

### Data analysis

The experimental data was obtained in the form avi videos which were broken up into single frames and converted into matrices using MATLAB. The mean of each matrix in the region of interest was calculated and normalized by the maximum mean measured throughout the time series sequence. Similarly, the results of the simulations were converted into a time series of normalized frames.

## Electronic supplementary material

Effect of Aspect ratio in the inner geometry configuration

Effect of Aspect ratio in the outer geometry configuration

Effect of Pulsatility on the inner 3.2 AR geometry configuration with water and glycerol solution at PI ranging from 0 to 2

Effect of curvature on the 3.2 AR geometry configuration

Supplementary Material
