# Supplementary material for: Mapping the Transport Kinetics of Molecules and Particles in Idealized Intracranial Side Aneurysms
Source: Sci Rep. 2018 Jun 4;8:8528. doi: 10.1038/s41598-018-26940-1 (PMC5986792; doi:10.1038/s41598-018-26940-1)
Supplement: Supplementary file 5 — Supplementary Material [file 41598_2018_26940_MOESM5_ESM.pdf]

Mapping the Transport Kinetics of Molecules and Particles in Idealized Intracranial Side Aneurysms

Supplementary Material

Mark Epshtein, Netanel Korin

(a) (b)

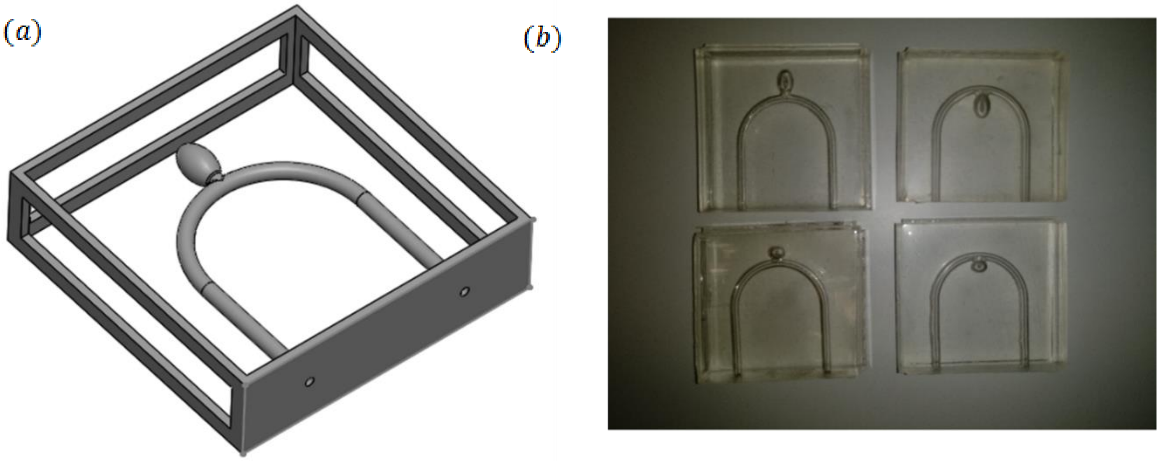

Figure S1 :Fabircation of aneurysm models. (a) CAD model of mold used for the fabrication of the silicone phantom of anerysm models (b) The obtained silicone models.

Model Production

A CAD model of the mold has been created in SolidWorks (Fig. S1a) and 3D printed from RDG720 plastic. This material breaks up in acetone into soft pieces without melting and sticking to the silicone thus allowing to remove to easily from the mold. The silicone (Sylguard 180) was degassed and poured into the mold, left to cure overnight and then placed in acetone for a few hours to remove the 3D printed plastic. The result is the phantom models, see for example Fig. S1b.

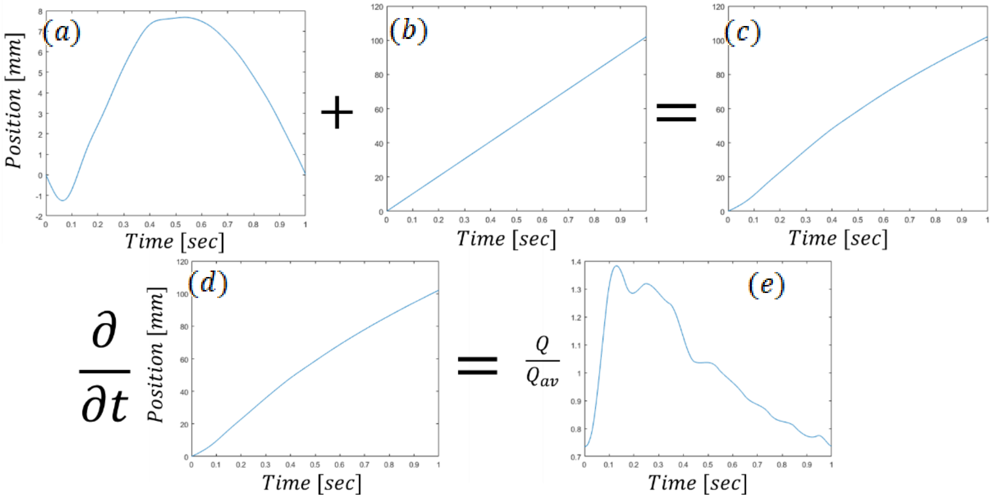

Figure S2: Schematic showing the desgin of the pulsatile flow waveform (a) motor position vs. the required time curve as calculated using equation 1 (b)The integration of the constant part gives the linear curve (c) The summation of both curve gives the position curve of the syringe required to produce the whole fluid displacement (d) and (e) the time derivative of this curve gives the desired flow waveform.

Pulsatile flow waveform production

A $1\,ml$ luer-lock syringe was connected to the oscillator linear motor. The motor's oscillations when combined with the constant flow of the pump were designed to produce the basilar artery waveform seen in Fig. S2. e[1]. Figure S2a is the motor position vs. time curve needed as calculated with equation 1 which is the time integration of the oscillatory part of the flow waveform: where Q_{av} is the average flow rate $100 - 200\ ml/min$ for the basilar artery A is the syringe cross-section and $Q(t)$ is the oscillatory part of the flowrate waveform and τ is a dummy variable. The integration of the constant part gives the linear curve in Fig. S2b (for illustration purpose).

$$D = \frac{Q_{av}}{A} \int_0^t \frac{Q(\tau)}{Q_{av}} d\tau \qquad (1)$$

The summation of both curves gives the position curve of the syringe as if it was producing the whole fluid displacement and the time derivative of this curve gives the desired waveform.

Glycerol solution viscosity measurement

The viscosity of a 40% glycerol solution was measured by a capillary tube viscometer (Cannon Fenske – reverse flow size 50).

Calibration for Self-Similarity of the results

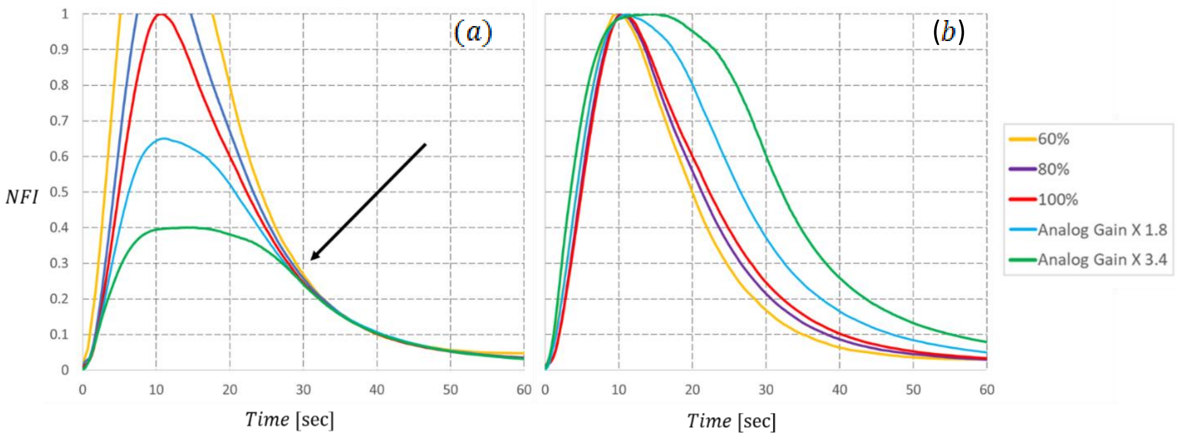

Figure S3: Self-Similarity of the measurements. (a) curves were multiplied by constants and above a certain time point (marked by the arrow) the curves coalesce (marked by the arrow) (b) fluorescence intensity curves of dye injection in the inner 3.2 AR model taken at different lighting and analog gain intensities conditions.

The bolus injection experiment is performed under an upright microscope, where the light is delivered to the top part of the model and the entire volume of the aneurysm is seen from above. In this mode of imaging, only the vertical projection of the bolus is seen while masking the mass underneath. Also, as the bolus is broken up, mixed and diluted inside the cavity its fluorescence falls, and when the light intensity is insufficient some information can become lost, primarily due to low fluorescence artifacts of diluted dye or particles. Figure S3b shows fluorescence intensity curves of the dye injection in the inner 3.2 AR model at different lighting and analog gain intensities conditions. It can be seen that as the intensity grows the curves become more stretched. This happens because the low intensity artifacts are seen longer. An analog gain only intensifies the measurements and saturates the camera thus the curve appears slightly cutoff.

However, Fig. S3.a shows that if the curves are multiplied by constants and forced to collapse into a single curve, above a certain time point (marked by the arrow) the curves will coalesce. This happens because the first part of the curve is convection dominated which removes the low fluorescence artifacts faster and appears differently for different intensities, while the second part is diffusion dominated which is a slow process and it would show the same results for different intensities if the intensity is high enough. If dye and particles show the same phenomena there exists a constant from which the curves collapse into each other, if on the other hand they show different phenomena the curves cannot be made to collapse. Since the curves cannot be brought to collapse in such a way, they must describe different processes. As a result of these investigations all our experiment have been set to the maximum possible intensity without the risk of saturation. For dye the setting was 100% intensity with no gain while for particle maximum gain was necessary.

Pulsatility results

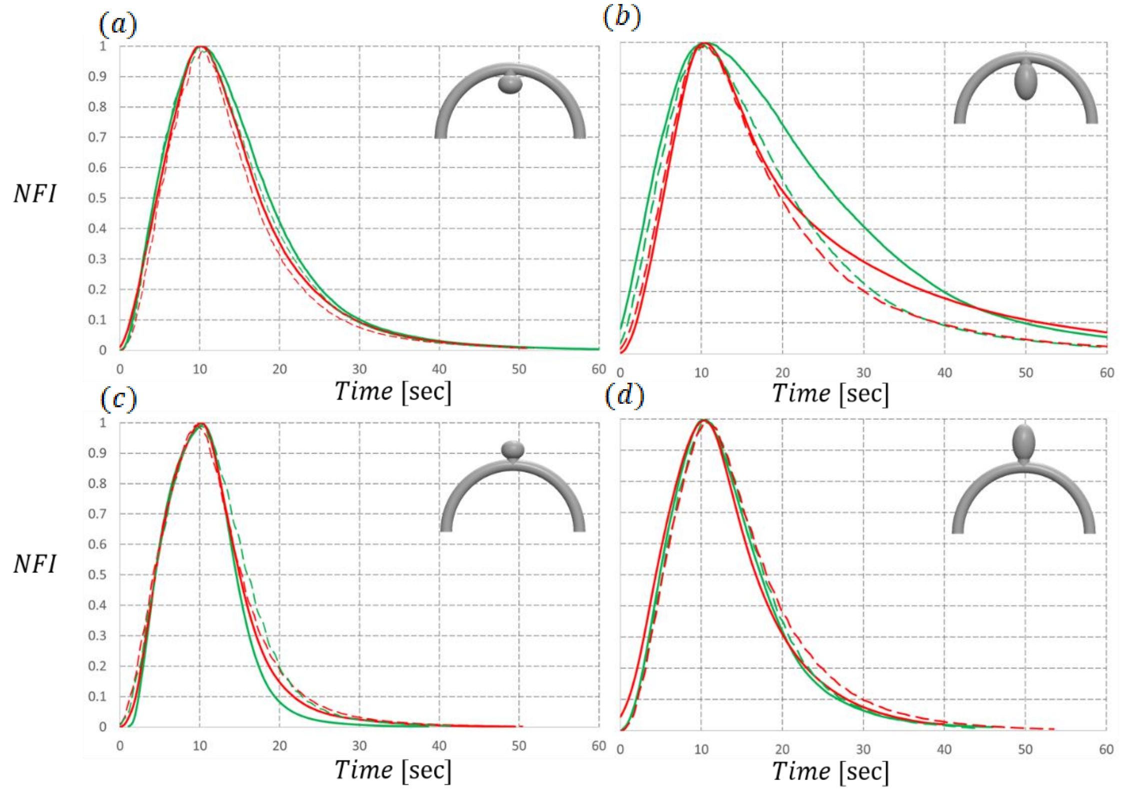

Figure S4: NFI curves of the pusatility results for all four geometries at PI=1 with water as flow medium, dashed line is pulsatile , red -particles and green for dye. (a) 1.6 IN (b) 3.2 IN (c) 3.2 [illegible]

Figure S4 shows the influence of pulsatility on the fluorescence intensity curves. The figure shows that when the medium is water, pulsatility brings the two curves together, this happens due to enhanced mixing at the aneurysm neck brought about by the retrograde flow of the Womersley profile which is more prominent in water than in a 40% glycerol solution because the Womersley number is reduce by half. The Womersely number (eq.2) is defined as the ratio between the transient inertial forces to viscous forces where L is a characteristic length-scale often chosen as the radius of the parent artery, ω is the angular frequency, ρ is the density of the medium and μ is the dynamic viscosity of the medium.

$$\alpha = L\sqrt{\frac{\omega\rho}{\mu}} \qquad (eq.2)$$

Thus, for water and 1 beat per second in the 3.2AR inner aneurysm the Womersley number with the current parent artery radius is 4.4 indicating a Womersley type flow profile in the pipe with retrograde flow at the aneurysm neck [2] increasing the viscosity four-folds will lower the chance of developing a Womersley flow profile and reducing mixing. Moreover, it has been shown that heat and mass transfer in areas of circulation is not affected much by pulsatility for Schmidt numbers over 1000[3]. The Schmidt number is defined as the ratio of viscous diffusion rate to mass diffusion rate (equation 3).

$$Sc = \frac{\mu}{\rho D} \qquad (eq.3)$$

Where μ is the dynamic viscosity, ρ is density and D is the mass diffusivity of the dye which is $4.25 \cdot 10^{-10}\, m^2/sec$ in water at room temperature. Thus, in our study $Sc = 2352$ for dye in water. Additionally, it is expected that due to the non-Newtonian shear-thinning behavior of real blood observed *in vivo,* blood viscosity inside cerebral aneurysms would be higher than in the parent arteries further increasing Sc. Thus, if the vortex is not broken by the pulsatility the mass transfer rate should not be much different than for constant flow. In the glycerol solution the diffusion of the dye is reduced increasing this number even further. Thus, the combination of the reduced Womersley number which keeps the vortex inside the aneurysm intact, and the increased Schmidt number are in agreement with the obtained results.

Results for 2,000,000 Da Dye

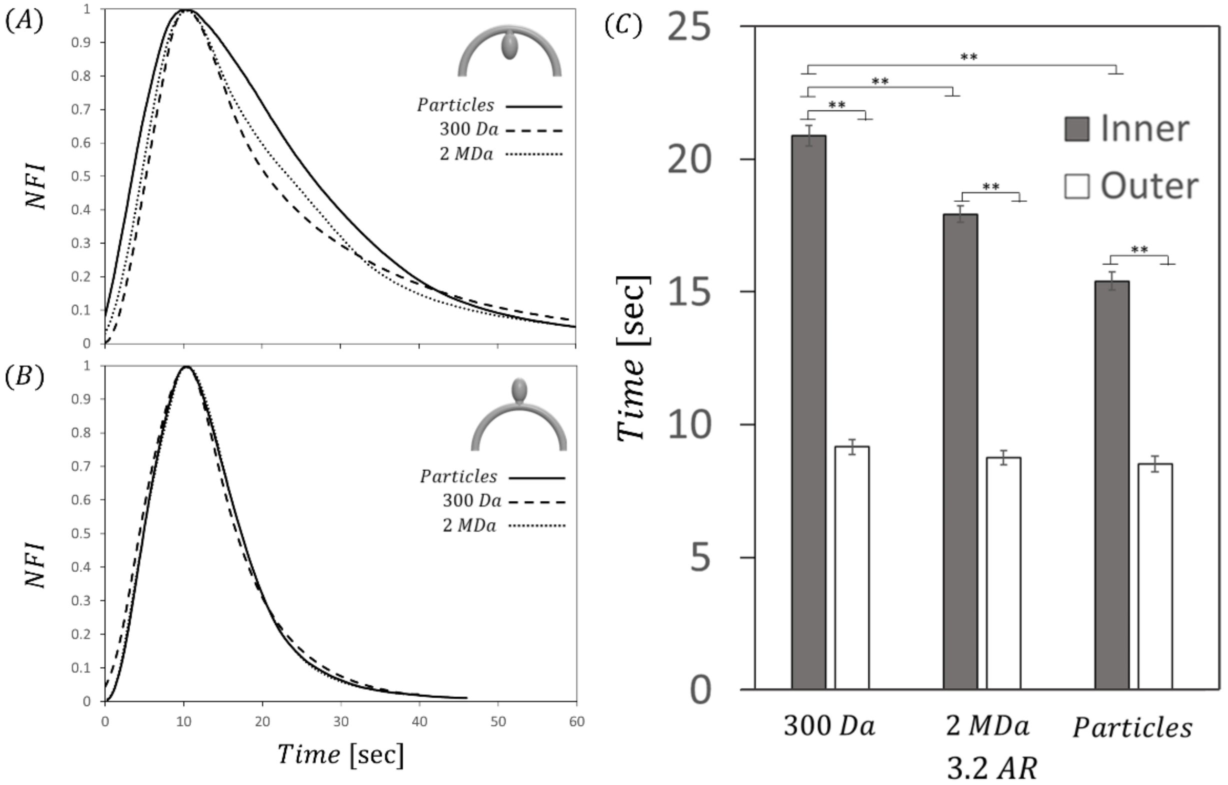

Figure S5: Experimental results showing mass transfer kinetics of a 2 MDa fluorescent dextran dye. (A) 3.2 AR inner geometry under constant flow where the 2 MDa dye can is the dotted curve (B) 3.2 AR outer geometry results showing the 2 MDa dye is indistinguishable from the 300 Da dye and particles results (C) Bar Graph showing time constants of bolus clearance for both inner and outer geometries, for the inner geometry the time constant is in between the 300 Da and particles constants. For the inner geometry, the result is statistically significant with P<0.02 the results for the outer geometry are not statistically different.

As a material that has a significantly lower diffusion rate compared to the 300 Da dye we used a 2,000,000 Dalton dye. The experiments were conducted with 2,000,000 Da Fluorescein isothiocyanate–dextran (52471 Sigma Aldrich) at 14 mg/100 ml, same as the concentration of the 300 Da dye described in previous experiments. As shown in Fig. 5S: for the 3.2 AR inner geometry the time constants are between the 300 Da dye and the particles suggesting that the difference between particles and 300 Da dye is large enough to include large and ultra-large molecules between these two cases. However, it showed be noted that by using dextran we

effectively changed the viscosity of the injected bolus thus adding another parameter to the system and inducing a two-phase flow. However, by the time the bolus reaches the aneurysm it is heavily diluted and its viscosity is close to the medium's viscosity. Nevertheless, we cannot accurately quantify these phenomena and thus further work is required to study this effect.

Effect of Gravity

The current study focused on horizontally imaged aneurysm thus discarding gravity effects. It should be noted, based on the Froude number, that gravity effects are not negligible and need to be tested in future works. The Froude number (eq.4) is defined as the ratio of flow inertia to the external field where u is velocity, g is the gravitational acceleration and l is a characteristic length scale.

$$Fr = \frac{u}{\sqrt{gl}} \qquad (eq. 4)$$

Figure S6 shows a volumetric rendering of the Froude number in a 3.2 AR inner geometry aneurysm where non-negligible areas of low Fr can be seen.

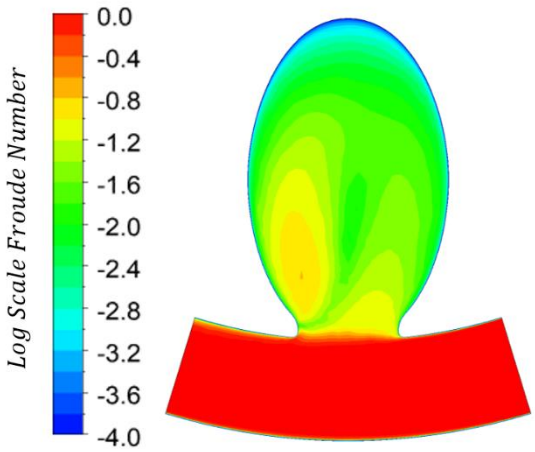

Figure S6: Froud number color bar map for a 3.2 AR inner aneurysm on the symmetry plane showing values lower than 1 that exists in the entire cavity.

Simulations

The equations solved were the laminar Naiver-Stokes equation (eq.5) coupled with the convection diffusion equation (eq.6) with no-slip condition assumed at the wall:

$$\rho\left(\frac{\partial \boldsymbol{u}}{\partial t} + u \cdot \nabla \boldsymbol{u}\right) = -\nabla p + \nabla \cdot \left(\mu(\nabla \boldsymbol{u} + (\nabla \boldsymbol{u})^T)\right) \qquad (5)$$

$$\frac{\partial c}{\partial t} = \nabla \cdot (D\nabla c)) - \nabla \cdot (\boldsymbol{u}c) \qquad (6)$$

where ρ is density, u is the velocity field, μ is the density, p is pressure, c is concentration and D is mass diffusivity.

The simulations were done in Ansys Fluent® and we used heat transfer equations as an analogy to mass transport. The results were normalized to produce the fluorescence curve. Mass concentration was converted to temperature and mass diffusivity was obtained by creating a material with heat conductivity, and heat capacity that satisfies equation 7. To obtain the corresponding heat conductivity the diffusivity ($\alpha = 4.25 \cdot 10^{-10} \frac{m^2}{sec}$ for fluorescein [4] and zero for particles) was multiplied by the fluid density (ρ) and the heat capacity (C_P) which were assumed the same as water

$$k = \alpha\rho C_p \qquad (7)$$

Since gravity was ignored, symmetry on one plane was assumed so the CAD models used for the constructions of the silicone models were cut along the parent artery and used also for the simulation. Also, the parent artery was truncated so that the radii of curvature are tangent to the aneurysm from both sides (see figure S7. A) it does not change the general direction of the flow as it follows the angle of the artery. The models were meshed using Ansys GAMBIT (the

difference between the CAD and the produced physical model is discussed below). 60,000 steps with a time steps between 0.001 sec for the inner geometries and 0.01 for the outer geometries were used to produce 60 second simulations with the SIMPLE solver. The elements used were tetrahedral and a five-layer inflation with 1.2 growth factor was used in all the models.

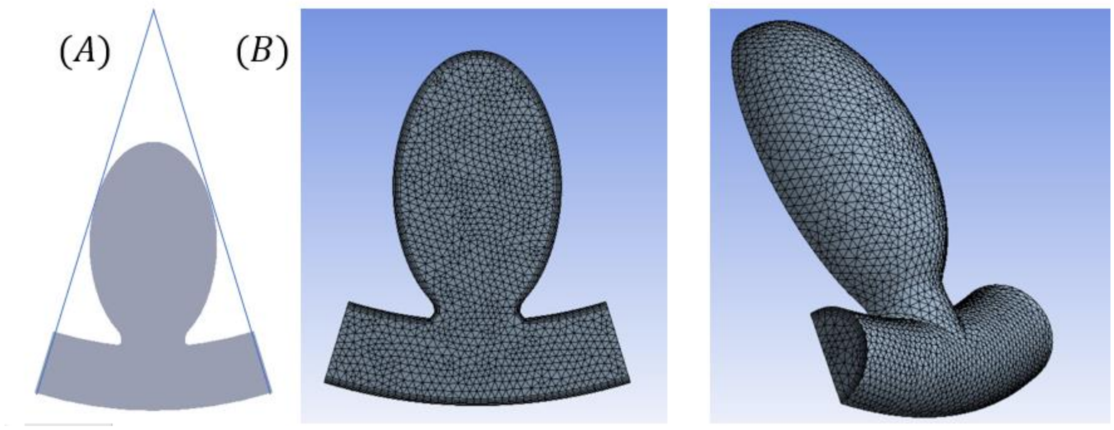

Figure S7: Illustration of the truncation radii for the 3.2 IN model (B) left: symmetry plane mesh for the 3.2 IN mod. Right: 3D view for the 3.2 IN mod.

The condition of all the simulations are summarized is table S1: Boundary conditions for all of the models were mass flow rate inlet and zero pressure outlet. Flow rate was similar to the experimental flow rate which maintained its Reynolds number. The chosen models were laminar flow coupled with the energy equation for heat transfer. No slip condition was maintained in all simulations.

Table S1 summary of simulation conditions

Geometry	***Cells***	***Time Step***	***Inlet***	***Outlet***	***Re***	***Laminar***	***No slip***
3.2 OU	120K	0.01 sec	50 ml/min	0 Pa	300	✓	✓
3.2 IN	240K	0.001 sec	50 ml/min	0 Pa	300	✓	✓
1.6 OU	120K	0.01 sec	50 ml/min	0 Pa	300	✓	✓
1.6 IN	120K	0.001 sec	50 ml/min	0 Pa	300	✓	✓

Post-processing

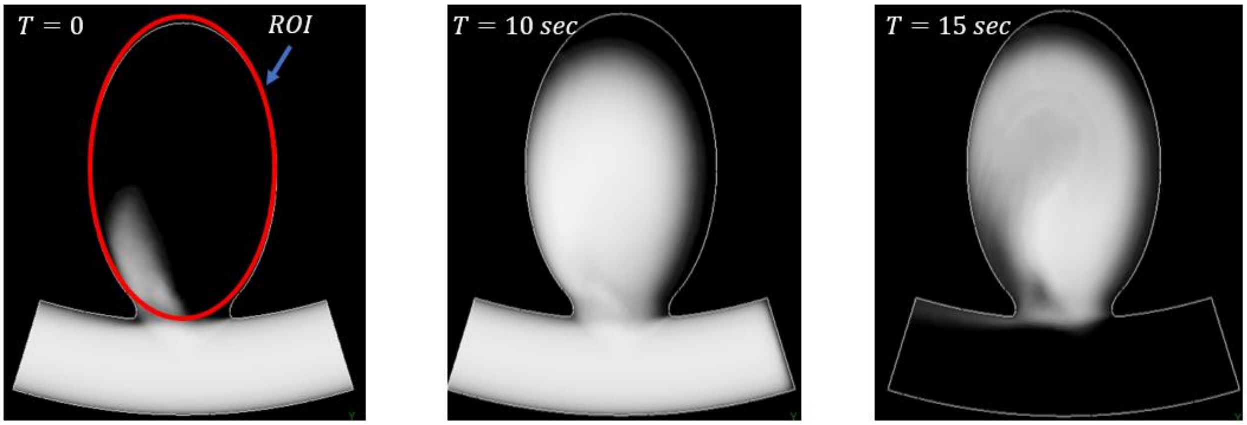

Figure S8 representative time series of simulation results for the 3.2 in geometry. Showing the volume rendering and region of interest.

The post-processing was done in Ansys CFD-POST. The simulations were presented in a volume rendering with 100 (CFD - post volume rendering option with 100 slices setting) of the temperature and analyze with the MATLAB program used to analyze the experiment videos (see Fig. S8) and MATLAB code below.

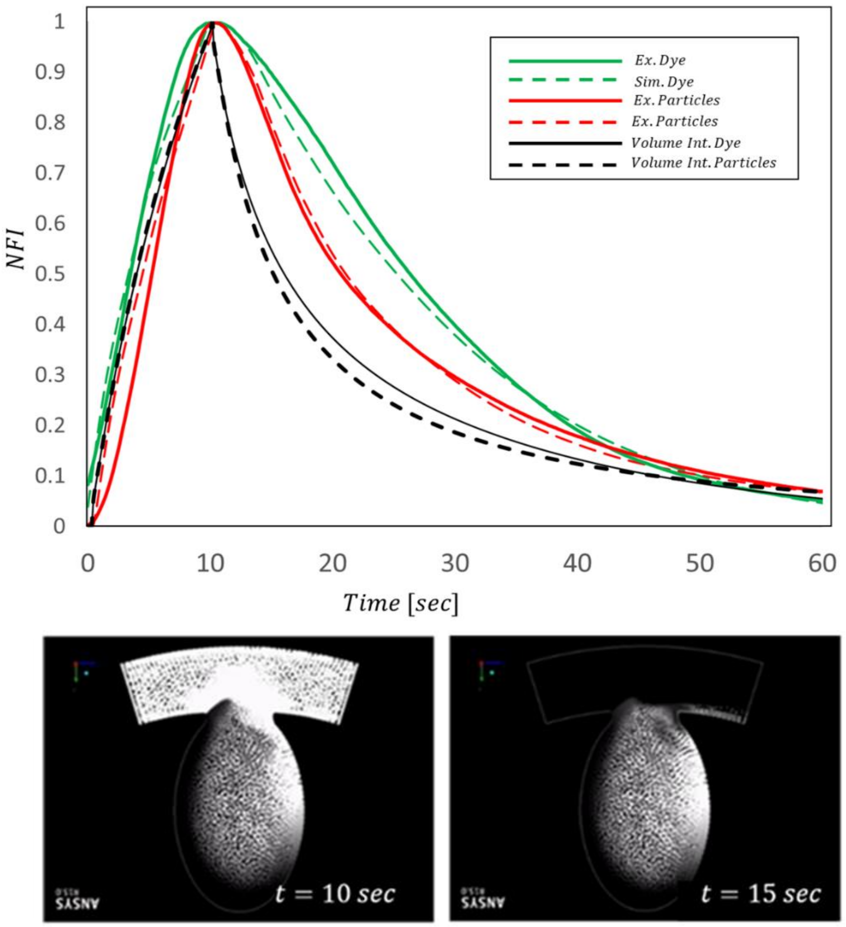

Figure S9 (A) NFI results for volume integration in the aneurysm domain where black dashed is for particles and black continues for dye showing siginfcnat difference compared to the utilized volume rendering method and experimental results where green is dye and red is particles while dashed is simulated and continuus is experimental. (B) Circular vector rendering of the concetration in the entire computational domain in a transparency scale ranging from transparent to white.

We performed also a volume integral of the dye/article concertation in the aneurysm domain and obtained the same trends regarding the curve inversion and particle lingering shown in fig. S9. A However, this method does not capture the masking of inner volume as happens when the entire volume is viewed from above. We thus opted for the volumetric rendering of the volume with a transparency color bar. To test whether visual artifact affect the results we visualized the volume both with volume rendering and circular vector representation and seen in figure S9. B we obtained similar results for both methods.

Convergence Criteria Selection

We have tested an order of magnitude smaller criteria and obtained the same results within less than 0.1%. Figure S10 shows the normalized maximum concentration (NC) in the entire computational domain for both the default convergence criteria and an order of magnitude smaller and appear identical.

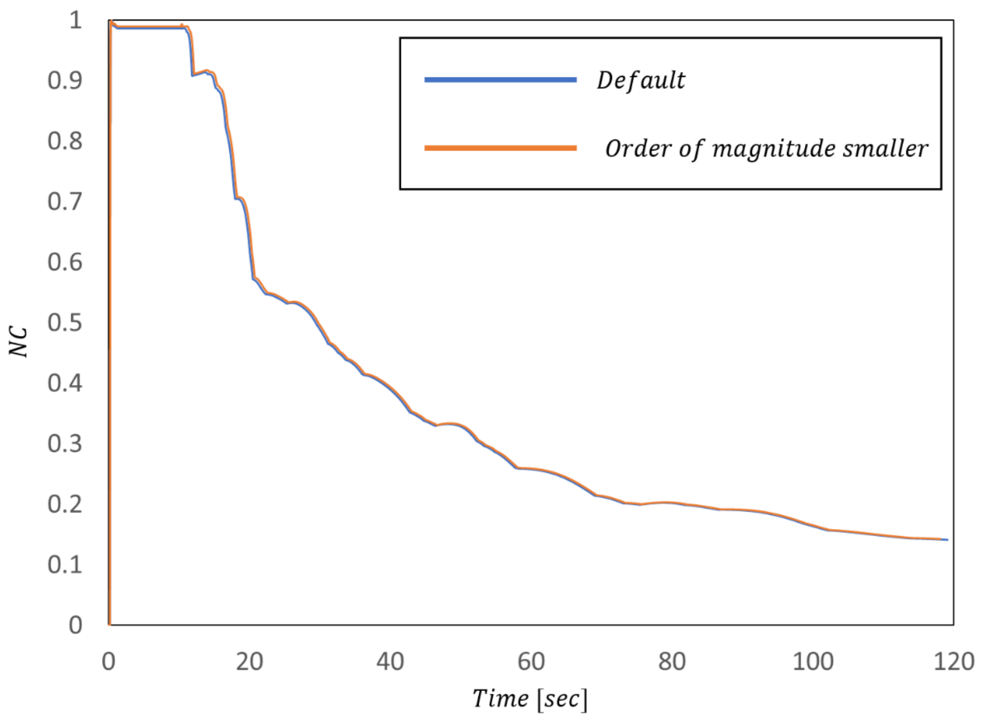

Figure S10 Simulation results for the normalized maximum concentration (NC) in the computation domain where blue is for the default convergence criteria and orange is an order of magnitude smaller criteria showing little differences between the curves (maximum difference 0.1%).

Spatial and temporal discretization

Table S2 the spatial and temporal discretization

**Spatial Discretization:**	**Transient Formulation:**
Gradient: Least Squares Cell Based<br>Pressure: Second Order<br>Momentum: Second Order Upwind<br>Energy: Second Order Upwind	First Order Implicit

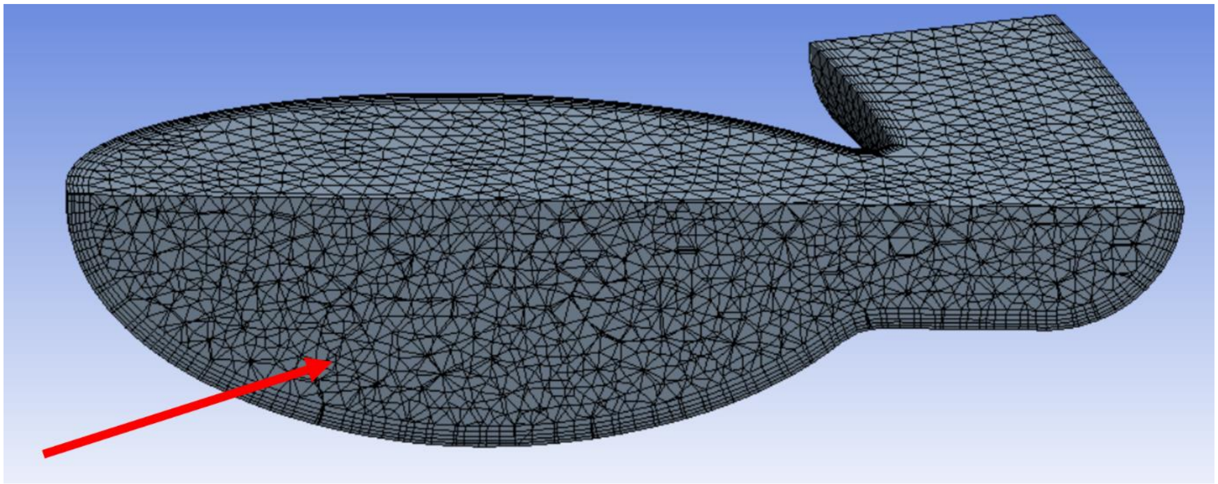

Figure S11 Illustration of the integration plane on the 3.2 inner geometry marked by the red arrow where concentration was integrated on the plane and the time curves used for mesh convergence investigation.

Mesh Convergence

For our purposes the coupling of diffusion and convection is highly important. Therefore, to test the mesh independence of our simulations we performed 15 second simulations (to capture the

entire bolus without completing the entire 60 second simulation) while integrating the concentration on the middle surface shown in Fig. S11 because every part of the bolus has to pass through it and the entire vortex passes through it. Three or two meshes were chosen for each of the four geometries each roughly double the cells of the next and time curves of the integrals on the surfaces were plotted, see Fig. S12. Two-time steps were tested for the chosen mesh 0.01 and 0.001 sec. It can be seen that the geometries were mesh and time step insensitive, the only geometry for which the finest mesh had to be chosen is the 3.2 AR inner configuration. Nevertheless, we chose the finest mesh out of the tested meshes for all of the cases.

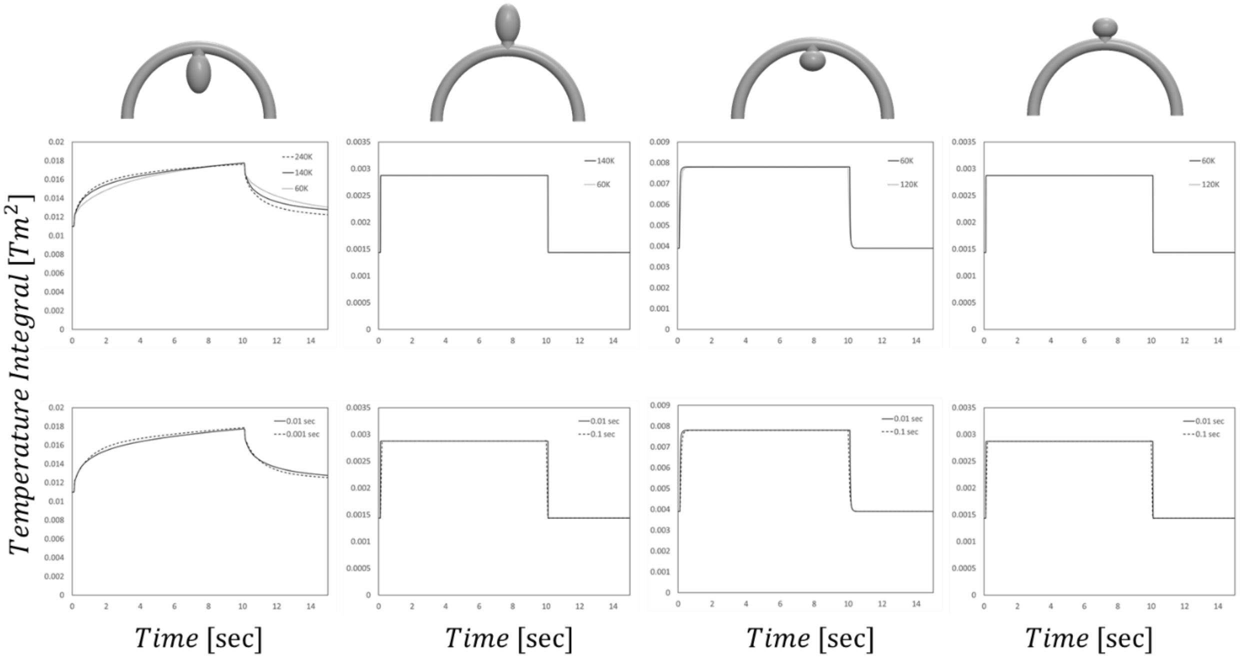

Figure S12 Top panel: Integration time curves for every mesh chosen and its corresponding geometry. Besides the 3.2 inner configuration, all geometries were insensitive to mesh size. Bottom panel: time curves for different times steps, all of the geometries were insensitive to the examined time step.

<u>Difference between the physical model and the CAD model</u>

The fabrication of the physical models inevitably produces some differences compared to the original CAD model. However, most of the difference are subtle except for the sharp corners which are prone to lacquer accumulation. We thus conducted simulations to examine the differences, as can be seen from Fig. S13 these are shown to be insignificant. We believe that this is because most of the mass transport occurs through the sides rather than the distal and proximal neck through a 3D “horse shoe “vortex inside the cavity, also the appendages only account for approximately 14% ($13.1\ vs\ 11.4\ mm^2$ original) increase in area which does not change the overall bulk flow characteristics through the cavity.

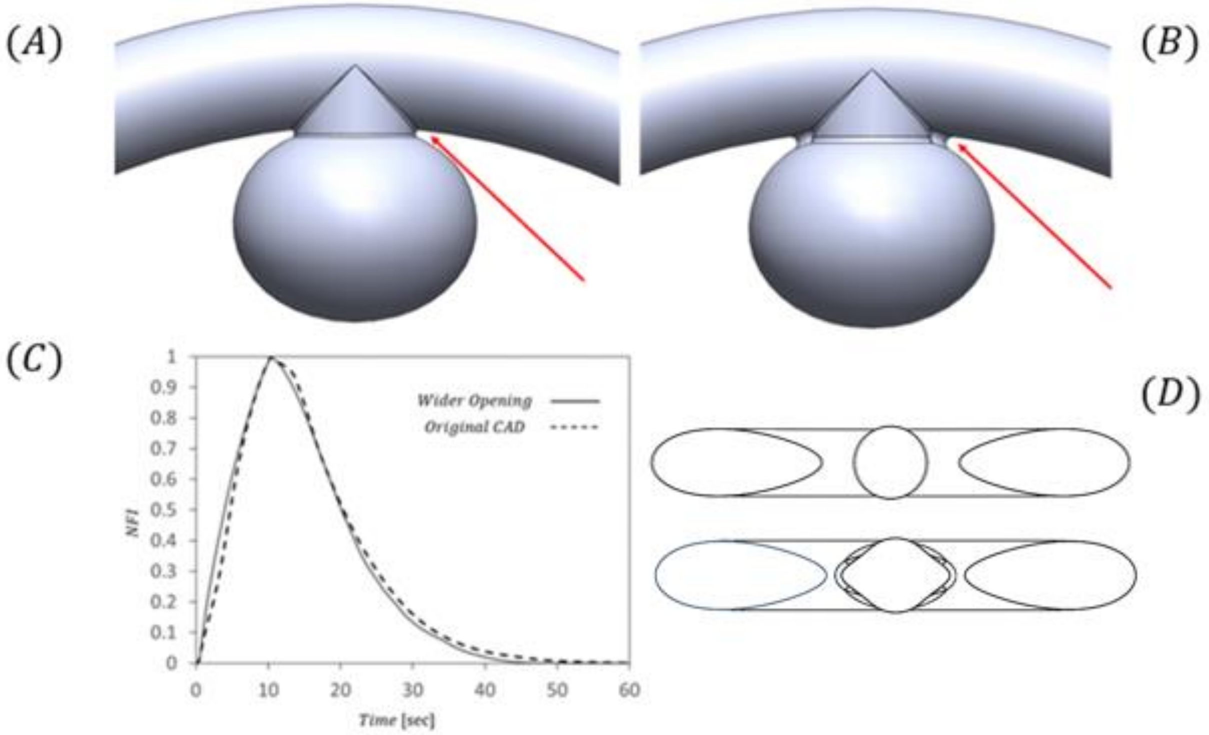

Figure S13 (A) original 1.6 IN CAD model that was used for physical model production (B) Model with appendages due to lacquer accumulation (C) simulation results for both models showing very good agreement (D) Aneurysm neck geometry showing the difference between the resulting neck geometries (upper with appendages lower: original).

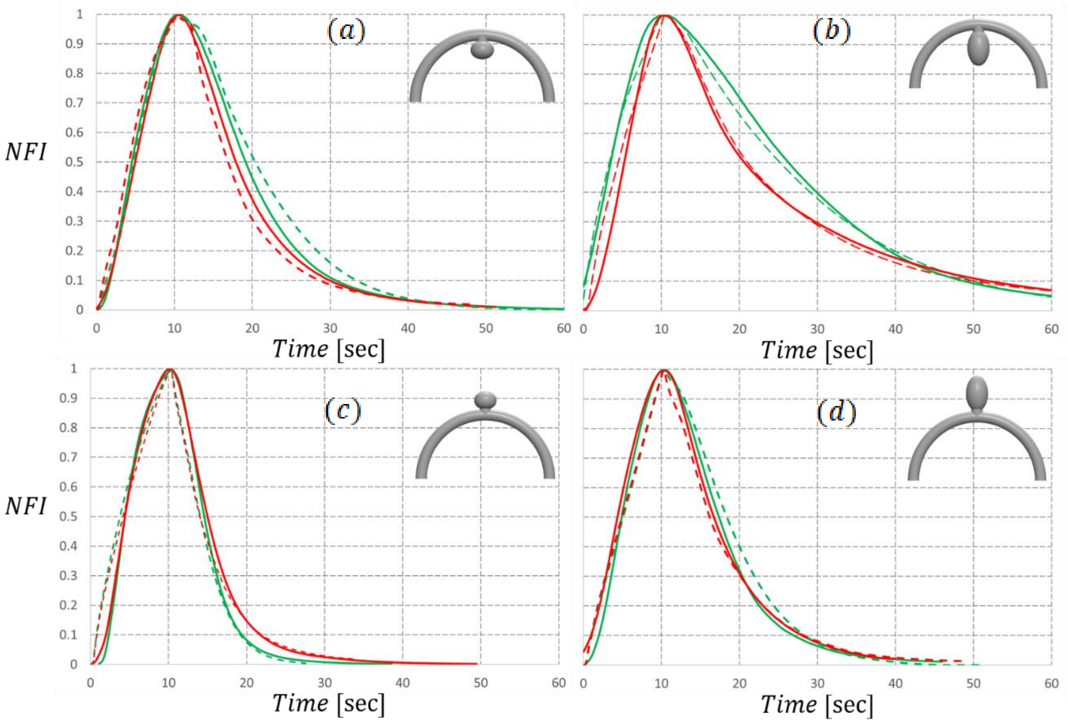

Figure S14 NFI curves of simulation results of all four geometries, dashed line is simulation and continuous is experiment, red -particles and green for dye. (a) 1.6 IN (b) 3.2 IN (c)1.6 OU (d)3.2 OU.

Simulation Results

The simulation results show good agreement with the experiments. Most importantly they show the same trends and effects brought about by the difference in diffusivity of the bolus.

References

1. Zhu, D. C., Xenos, M., Linninger, A. A. & Penn, R. D. Dynamics of lateral ventricle and cerebrospinal fluid in normal and hydrocephalic brains. *J. Magn. Reson. Imaging* **24,** 756–770 (2006).

2. Hale, J. F., McDonald, D. A. & Womersley, J. R. Velocity profiles of oscillating arterial flow, with some calculation of viscous drag and the Reynolds number. *J. Physiol.* **128,** 629–640 (1955).

3. Ma, P., Li, X. & Ku, D. N. Heat and mass transfer in a separated flow region for high Prandtl and Schmidt numbers under pulsatile conditions. *Int. J. Heat Mass Transf.* **37,** 2723–2736 (1994).

4. Kapusta, P. Absolute diffusion coefficients: compilation of reference data for FCS calibration. *Appl. note* 0–1 (2010).

MATLAB

```
%% Obtain Frames from video

obj = VideoReader('2.mp4');
numberOfFrames = obj.NumberOfFrames;
frames = obj.NumberOfFrames;
for x = 1 :frames
    vid = read(obj,x);
    imwrite(vid(:,:,:,1),strcat('fl-',num2str(x),'.tif'));
end
```

%% set ROI

```
A= imread(strcat('fl-',num2str(50),'.tif'));
A=imgaussfilt(A, 2);
figure, imshow(A);
h = imellipse(gca,[396 311.5 492 367]);
addNewPositionCallback(h,@(p) title(mat2str(p,3)));
fcn =
makeConstrainToRectFcn('imellipse',get(gca,'XLim'),get(gca,'YLim
'));
setPositionConstraintFcn(h,fcn);
```

%% use ROI to calculate average fluorescence intensity in the ROI

```
figure('Visible', 'off');

n=1;
for x = 1 : frames
A= imread(strcat('fl-',num2str(x),'.tif'));

h_im = image(A,'Visible', 'off');
e = imellipse(gca,[396 311.5 492 367]);
BW = createMask(e,h_im);
BW(:,:,2) = BW;
BW(:,:,3) = BW(:,:,1);
```

```
ROI = A;

ROI(BW == 0) = 0;

imshow(ROI);

r(n)=mean(mean(ROI(:)));

        n=n+1;

end
```

%% Normalize FI curves

```
Cut=10;Rn=10; %Frames per second and cut frames before bolus
reaches the cavity

rt=(r-min(r))/(max(r)-min(r));

rm=(rt(Cut:end)-min(rt(Cut:end)))/(max(rt(Cut:end))-
min(rt(Cut:end)));

figure ('color' , [1 1 1]);

time=linspace(0,frames/Rn,length(r(Cut:end)));

hold on


plot(time,rm);

xlim([0 120])

xlabel ('time [sec]');

ylabel ('%');
```
